# Switching to angiotensin receptor–neprilysin inhibitor and reduced risk of new-onset atrial fibrillation in heart failure with reduced ejection fraction: a nationwide propensity score–matched study

**DOI:** 10.1093/ehjcvp/pvag074

**Published:** 2026-09-15

**Authors:** Young Jun Park, Dong-Hyuk Cho, Jimi Choi, Jong-Chan Youn, Mi-Na Kim, Chan Joo Lee, Jung-Woo Son, Byung-Su Yoo

**Affiliations:** Division of Cardiology, Department of Internal Medicine, Korea University Anam Hospital, Korea University College of Medicine, Seoul 02841, South Korea; Division of Cardiology, Department of Internal Medicine, Korea University Anam Hospital, Korea University College of Medicine, Seoul 02841, South Korea; Division of Endocrinology and Metabolism, Department of Internal Medicine, Korea University Anam Hospital, Korea University College of Medicine, Seoul 02841, South Korea; Division of Cardiology, Department of Internal Medicine, Seoul St. Mary’s Hospital, Catholic Research Institute for Intractable Cardiovascular Disease, College of Medicine, Catholic University of Korea, Seoul 06591, South Korea; Division of Cardiology, Department of Internal Medicine, Korea University Anam Hospital, Korea University College of Medicine, Seoul 02841, South Korea; Division of Cardiology, Department of Internal Medicine, Severance Hospital, Yonsei University College of Medicine, Seoul 03722, South Korea; Division of Cardiology, Department of Internal Medicine, Yonsei University Wonju College of Medicine, Wonju 26426, South Korea; Division of Cardiology, Department of Internal Medicine, Yonsei University Wonju College of Medicine, Wonju 26426, South Korea

**Keywords:** Angiotensin receptor–neprilysin inhibitor, Atrial fibrillation, Heart failure with reduced ejection fraction, Renin–angiotensin system blockade, Real-world evidence

## Abstract

**Aims:**

We investigated whether switching from traditional renin–angiotensin system (RAS) blockade to angiotensin receptor–neprilysin inhibitor (ARNI) is associated with a lower risk of new-onset atrial fibrillation (AF) than continuing RAS blockade in patients with heart failure with reduced ejection fraction (HFrEF) without prior AF.

**Methods and results:**

Using data from the Korean National Health Insurance Service (2017–2021), we identified 39 061 patients with HFrEF without prior AF who received either ARNI (*n* = 12 263) or traditional RAS blockade (*n* = 26 798). After 1:1 propensity score matching, 9030 patients were included in each group. The primary outcome was new-onset AF within 1 year. In the propensity score–matched cohort, new-onset AF incidence was lower in the ARNI group [10.73 vs. 12.15 per 100 person-years; hazard ratio (HR) 0.89; 95% confidence interval (CI) 0.81–0.98; *P* = 0.021]. The composite outcome (new-onset AF, ischaemic stroke, or all-cause death) was also reduced (HR 0.85; 95% CI 0.79–0.92; *P* < 0.001). In competing risk analysis, ARNI was associated with a lower subdistribution hazard of new-onset AF (sHR 0.73; 95% CI 0.58–0.91; *P* = 0.005). In the unmatched cohort, the HR was 0.85 (95% CI 0.75–0.96) in an exploratory model including age, sex, hypertension, chronic kidney disease, prior ischaemic stroke, socioeconomic status, residential region, and beta-blocker use, and 0.94 (95% CI 0.87–1.02) after adjustment for all measured baseline covariates.

**Conclusion:**

In this large nationwide cohort of AF-naive patients with HFrEF, switching to ARNI was associated with a lower risk of short-term new-onset AF than continuing traditional RAS blockade. As this was a retrospective study, further prospective studies are needed to confirm these findings.

## Graphical Abstract

**Graphical Abstract:**
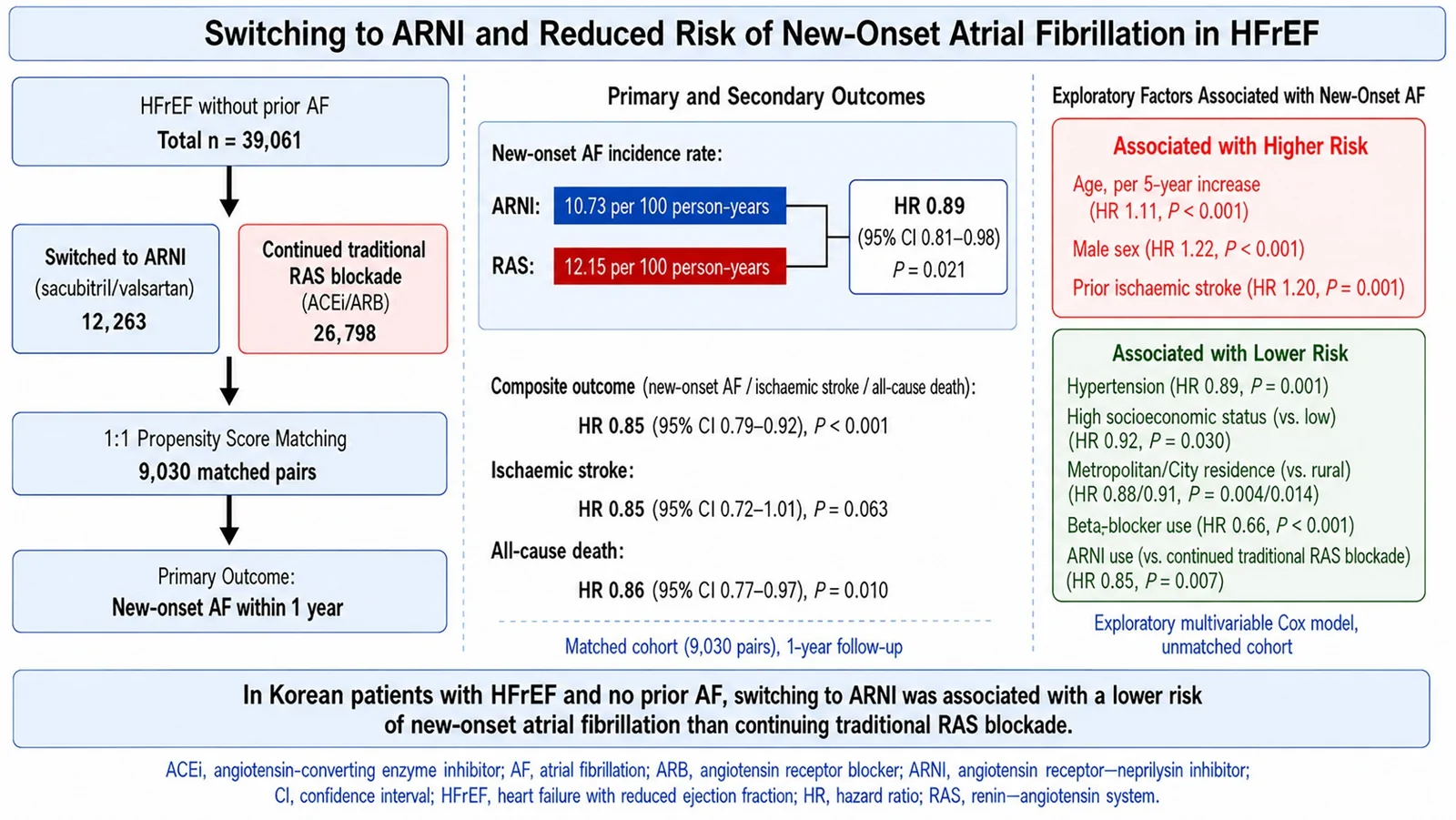
Switching to angiotensin receptor–neprilysin inhibitor and reduced risk of new-onset atrial fibrillation in heart failure with reduced ejection fraction. Left panel: Study design showing the cohort of 39 061 patients with HFrEF without prior AF, switching to ARNI or continuing traditional RAS blockade, 1:1 propensity score matching (9030 pairs), and the primary outcome. Middle panel: Incidence rates and hazard ratios for new-onset AF and secondary outcomes in the propensity score–matched cohort. Right panel: Exploratory factors associated with new-onset AF in a multivariable Cox model in the unmatched cohort, including factors associated with higher risk (age per 5-year increase, male sex, and prior ischaemic stroke) and lower risk (hypertension, high socioeconomic status, metropolitan or city residence, beta-blocker use, and ARNI use). ACEi, angiotensin-converting enzyme inhibitor; AF, atrial fibrillation; ARB, angiotensin receptor blocker; ARNI, angiotensin receptor–neprilysin inhibitor; CI, confidence interval; HFrEF, heart failure with reduced ejection fraction; HR, hazard ratio; RAS, renin–angiotensin system.

## Introduction

Atrial fibrillation (AF) is the most common sustained cardiac arrhythmia, frequently coexisting with heart failure (HF), and the two conditions share common pathophysiological mechanisms that perpetuate each other.^1,2^ The development of new-onset AF in patients with HF is associated with worsened haemodynamic status, increased risk of thromboembolism, and higher mortality.^3,4^ In patients with HF with reduced ejection fraction (HFrEF), the incidence of new-onset AF is particularly high, with reported rates of up to 10%–15% per year.^5^ Thus, prevention of new-onset AF in patients with HFrEF represents an important therapeutic target.

Angiotensin receptor–neprilysin inhibitor (ARNI), a combination of sacubitril and valsartan, has become a cornerstone of HFrEF management. ARNI reduced cardiovascular mortality and HF hospitalization compared with enalapril in the PARADIGM-HF trial,^6^ and is now recommended as first-line therapy for HFrEF.^7,8^ In addition to its haemodynamic effects, ARNI augments natriuretic peptide signalling, which may lower left atrial pressure and attenuate atrial fibrosis and dilatation,^9,10^ providing a mechanistic basis for AF prevention.

However, evidence regarding the AF-preventive effect of ARNI in HFrEF remains limited. In the PARADIGM-HF trial, new-onset AF showed no significant difference between groups.^6^ Similarly, meta-analyses of RCTs have not demonstrated a significant AF reduction with ARNI, although limited by small event numbers and heterogeneous populations.^11,12^ Large-scale real-world evidence accounting for competing risks of mortality is needed to define the AF-preventive role of ARNI.

Using the Korean National Health Insurance Service (NHIS) database, we investigated whether switching from traditional RAS blockade to ARNI is associated with a lower risk of new-onset AF than continuing RAS blockade in patients with HFrEF without prior AF.

## Methods

### Data source

This nationwide population-based cohort study utilized data from the Korean NHIS Database. The Korean NHIS is a single, mandatory medical insurer managed by the Korean Ministry of Health, Welfare, and Family Affairs, covering 97% of the Korean population. The database encompasses demographic and anthropometric data, pharmaceutical dispensing records, and administrative claims coded in accordance with the International Statistical Classification of Diseases and Related Health Problems, 10th Revision (ICD-10). Mortality information was sourced from Statistics Korea. The database structure and methodology have been previously described.^13^ Access to the National Health Insurance Service (NHIS) database was granted under a memorandum of understanding between the Korean Society of Cardiology and the NHIS (database number: NHIS-2022-1-376). The study was approved by the Institutional Review Board of Yonsei University Wonju Severance Christian Hospital (approval number: CR321358). The study complied with the Declaration of Helsinki and was conducted and reported in accordance with the Strengthening the Reporting of Observational Studies in Epidemiology guidelines. The requirement for individual informed consent was waived, as the NHIS database contains only de-identified data assembled in accordance with strict confidentiality regulations.

### Study patients

The study population comprised patients with HFrEF and no prior history of AF who newly initiated ARNI or were receiving traditional RAS blockade between July 2017 and December 2021. HFrEF was defined using two complementary criteria: (i) prescription of ARNI, given that the Korea Health Insurance Review and Assessment Service restricts ARNI reimbursement to patients with left ventricular ejection fraction ≤40%; or (ii) two or more diagnostic claims for any of the following ICD-10 codes: HF with left ventricular systolic dysfunction (I50.04), left ventricular failure (I50.1), dilated cardiomyopathy (I42.0), or ischaemic cardiomyopathy (I25.5), combined with at least one natriuretic peptide measurement or echocardiographic examination within 6 months of the prescription date. Traditional RAS blockade comprised angiotensin-converting enzyme (ACE) inhibitors and angiotensin receptor blockers (ARBs); the individual agents are provided in Supplementary material online, *Table S1*.

A 90-day exposure ascertainment period was applied after the first qualifying prescription. During this period, patients in the ARNI group were required to receive continuous ARNI treatment for 90 days without any interruption exceeding 30 days, and patients in the RAS blockade group were required to receive continuous RAS blockade for 90 days without any interruption exceeding 30 days; patients in the RAS blockade group did not subsequently receive ARNI during the study period. The index date was defined as 90 days after the first qualifying prescription, and follow-up for study outcomes began at the index date.

The following exclusion criteria were applied: age younger than 18 years, diagnosis of cancer within 5 years, end-stage renal disease requiring dialysis, prior heart transplantation or implantation of a left ventricular assist device, human immunodeficiency virus infection, nursing-home admission at the index date, no prior use of traditional RAS blockade before the index date, a prescription duration for ARNI or traditional RAS blockade of fewer than 90 days, and a documented prior history of AF. New-onset AF was defined as at least one inpatient claim or at least two outpatient claims of ICD-10 code I48, a validated operational definition for AF in the Korean National Health Information Database.^14^ Following application of these criteria, 12 263 patients were assigned to the ARNI group and 26 798 to the RAS blockade group (*Figure 1*).

**Figure 1:**
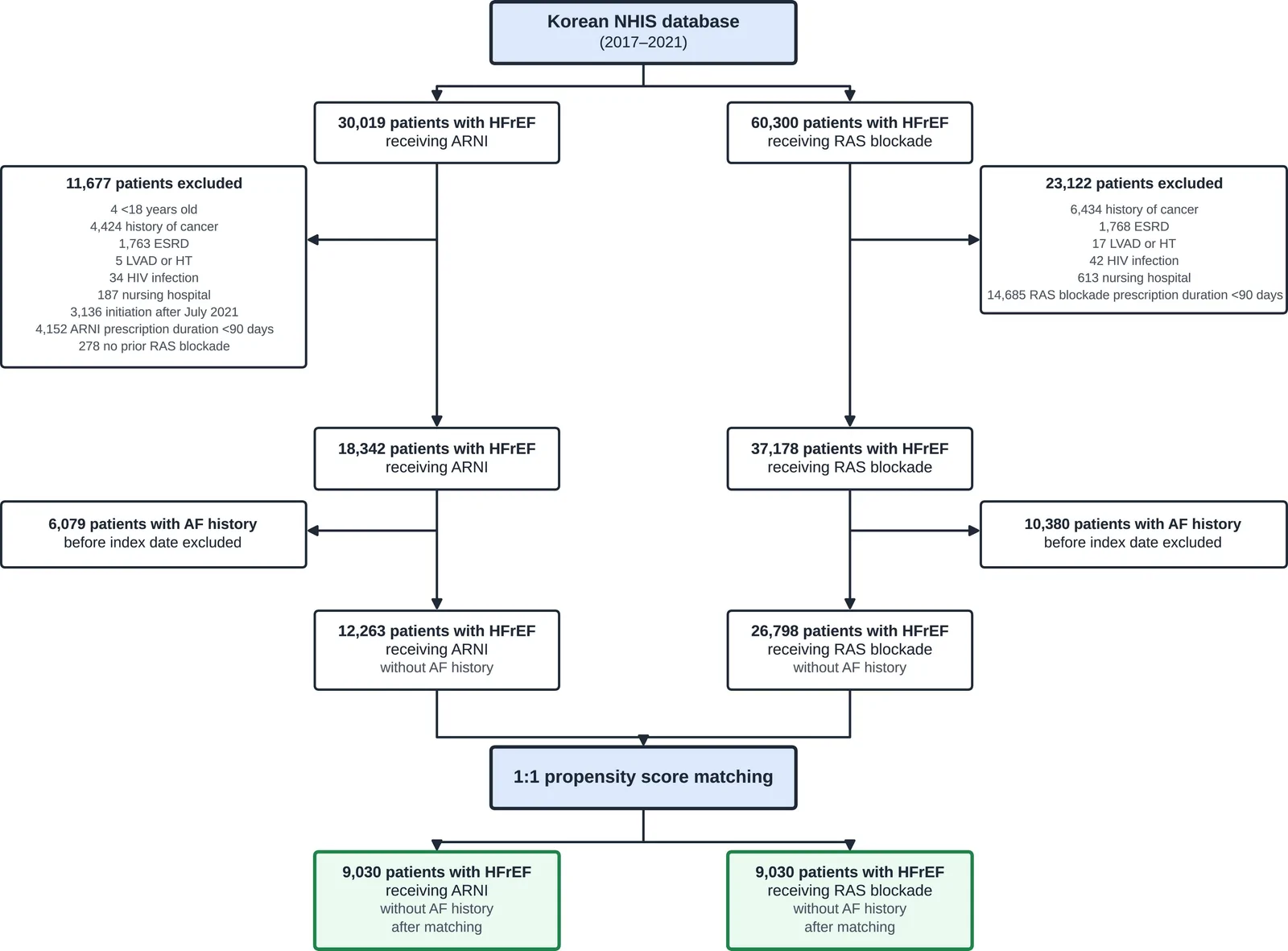
Schematic of study flow. Flow diagram of patients with HFrEF identified in the Korean National Health Insurance Service database from 2017 to 2021. Sequential application of the eligibility criteria yielded 12 263 patients who switched from ACE inhibitor or angiotensin receptor blocker therapy to ARNI and 26 798 patients who continued ACE inhibitor or angiotensin receptor blocker therapy, all without documented prior AF. After 1:1 propensity-score matching, 9030 patients were included in each group. ACE, angiotensin-converting enzyme; AF, atrial fibrillation; ARNI, angiotensin receptor–neprilysin inhibitor; ESRD, end-stage renal disease; HFrEF, heart failure with reduced ejection fraction; HIV, human immunodeficiency virus; HT, heart transplantation; LVAD, left ventricular assist device; NHIS, National Health Insurance Service; RAS, renin–angiotensin system.

### Study endpoints

The primary outcome was new-onset AF within 1 year, defined as at least one inpatient claim or at least two outpatient claims of ICD-10 code I48. Secondary outcomes included ischaemic stroke, all-cause death, and a composite of new-onset AF, ischaemic stroke, or all-cause death within 1 year. Ischaemic stroke was defined using ICD-10 codes I63–I64 during hospitalization with brain imaging within 7 days of admission. All-cause mortality was obtained from Statistics Korea. Each outcome was assessed from the index date until the occurrence of the outcome of interest, death, loss to follow-up, or the end of the study period (31 December 2021), whichever came first.

### Statistical analysis

Between-group differences in demographic and clinical characteristics were quantified using absolute standardized differences (ASDs). Propensity scores were estimated using multivariable logistic regression, with treatment assignment (ARNI vs. traditional RAS blockade) as the dependent variable and all baseline characteristics shown in *Table 1* as covariates, including demographic factors, income-based socioeconomic status, residential region, individual comorbidities, the Charlson comorbidity index, concomitant cardiovascular medications, healthcare utilization variables, and the duration of prior RAS blockade. Patients were matched 1:1 by greedy nearest-neighbour matching without replacement, using a calliper of 0.1. Index year was matched exactly during the matching process, so that paired patients entered the cohort in the same calendar year. The propensity-score model showed adequate discrimination, with a c-statistic of 0.72. An ASD less than 0.1 was considered evidence of satisfactory covariate balance, and all covariates met this threshold (*Table 1*). After matching, 9030 patients were selected for each group.

**Table 1:** Baseline characteristics of study groups

	Before matching			After matching^a^		
	RAS blockade (*n* = 26 798)	ARNI (*n* = 12 263)	ASD	RAS blockade (*n* = 9030)	ARNI (*n* = 9030)	ASD
Age	69.0 ± 14.8	66.0 ± 14.0	0.207	67.4 ± 14.8	67.4 ± 13.8	0.004
Women	12 328 (46.0)	4191 (34.2)	0.243	3486 (38.6)	3494 (38.7)	0.002
SES			0.049			0.049
Low	8464 (31.6)	3947 (32.2)		2670 (29.6)	2863 (31.7)	
Middle	8266 (30.8)	3999 (32.6)		2921 (32.3)	2888 (32.0)	
High	10 068 (37.6)	4317 (35.2)		3439 (38.1)	3279 (36.3)	
Region			0.154			0.054
Metropolitan	4592 (17.2)	2189 (17.9)		1471 (16.3)	1587 (17.6)	
City	5956 (22.2)	3410 (27.8)		2314 (25.6)	2291 (25.4)	
Rural	16 250 (60.6)	6664 (54.3)		5245 (58.1)	5152 (57.0)	
Burden of comorbidities						
CCI, median (IQR)	4 (2–5)	4 (2–5)	0.018	4 (2–5)	4 (2–5)	0.016
Diabetes mellitus	6157 (23.0)	3690 (30.1)	0.162	2483 (27.5)	2526 (28.0)	0.011
Hypertension	18 383 (68.6)	9394 (76.6)	0.180	6746 (74.7)	6748 (74.7)	0.001
Dyslipidaemia	13 370 (49.9)	7412 (60.4)	0.213	5206 (57.7)	5220 (57.8)	0.003
IHD	15 037 (56.1)	8063 (65.8)	0.199	5705 (63.2)	5728 (63.4)	0.005
Cerebrovascular disease	2660 (9.9)	1019 (8.3)	0.056	805 (8.9)	781 (8.7)	0.009
Ischaemic stroke	2206 (8.2)	902 (7.4)	0.033	693 (7.7)	684 (7.6)	0.004
PAD	791 (3.0)	350 (2.9)	0.006	271 (3.0)	260 (2.9)	0.007
CKD	1961 (7.3)	1031 (8.4)	0.041	750 (8.3)	757 (8.4)	0.003
Medication						
SGLT2 inhibitor	876 (3.3)	891 (7.3)	0.180	487 (5.4)	512 (5.7)	0.012
CCB	9699 (36.2)	2531 (20.6)	0.350	2213 (24.5)	2253 (25.0)	0.010
Aldosterone antagonist	6712 (25.1)	6379 (52.0)	0.577	3770 (41.8)	3758 (41.6)	0.003
Diuretics except aldosterone antagonist	14 489 (54.1)	8989 (73.3)	0.408	6179 (68.4)	6236 (69.1)	0.014
Beta-blocker	15 700 (58.6)	9227 (75.2)	0.360	6264 (69.4)	6334 (70.1)	0.017
Digoxin	1823 (6.8)	1259 (10.3)	0.124	832 (9.2)	849 (9.4)	0.007
Statin	14 463 (54.0)	7058 (57.6)	0.072	5152 (57.1)	5097 (56.5)	0.012
Healthcare utilization						
Duration of RAS blockade before index date (months)	46.0 ± 48.4	38.5 ± 47.0	0.157	42.3 ± 46.7	41.8 ± 49.0	0.012
No. of outpatient clinic visits, median (IQR)	22 (12–39)	21 (12–36)	0.053	22 (11–38)	21 (12–37)	0.010
Hospitalization within 1 year before index date	14 844 (55.4)	7654 (62.4)	0.143	5256 (58.2)	5295 (58.6)	0.009
No. of hospitalizations, median (IQR)	1 (1–2)	1 (1–2)	0.012	1 (1–2)	1 (1–2)	0.008
Duration of hospitalization (days)	6 (1–14)	8 (3–17)	0.171	7 (2–15)	8 (3–17)	0.061
ED visit	469 (1.8)	297 (2.4)	0.047	201 (2.2)	195 (2.2)	0.005

Data are expressed as frequencies (percentages), means ± standard deviations, and medians (interquartile ranges).

ARNI, angiotensin receptor–neprilysin inhibitor; ASD, absolute standardized difference; CCB, calcium channel blocker; CCI, Charlson comorbidity index; CKD, chronic kidney disease; ED, emergency department; IHD, ischaemic heart disease; IQR, interquartile range; PAD, peripheral artery disease; RAS, renin–angiotensin system; SES, socioeconomic status; SGLT2, sodium-glucose co-transporter-2.

^a^Propensity scores were estimated using multivariable logistic regression, including all baseline covariates presented in this table. Patients were subsequently matched 1:1 using greedy nearest-neighbour matching without replacement, with a calliper of 0.1 and exact matching on index year.

The primary analysis followed an intention-to-treat-like principle, classifying patients according to their treatment at the index date and retaining them in that group throughout follow-up, regardless of subsequent discontinuation or changes in therapy. The incidence rates of outcomes were calculated as the number of events per 100 person-years. A Cox proportional hazards regression model stratified by index year was used to evaluate the association between treatment groups and study outcomes. For analyses conducted before matching, two multivariable-adjusted Cox regression models were fitted. Model A (all-baseline-covariate–adjusted model) included all variables listed in *Table 1*. Model B (PARADE-HF–specified model) followed the adjustment strategy used in the parent PARADE-HF study and included covariates with an absolute standardized difference ≥0.1 before propensity score matching in the parent cohort: age, sex, residential region, diabetes mellitus, hypertension, dyslipidaemia, ischaemic heart disease, chronic kidney disease, use of sodium-glucose cotransporter 2 inhibitors, calcium channel blockers, aldosterone antagonists, diuretics, and beta-blockers, duration of prior RAS blockade, and hospitalization within 1 year before the index date.^15^ Prior AF also met this threshold in the parent cohort but was not included because the present cohort was restricted to patients without prior AF. For analyses in the propensity score–matched cohort, we estimated hazard ratios (HRs) using a Cox proportional hazards model with treatment group as the sole covariate and a robust sandwich variance estimator clustered by matched pair to account for the within-pair correlation induced by propensity score matching.^16^ To account for the competing risk of death, we performed subdistribution hazard regression using the Fine–Gray method.^17^ In addition, an exploratory multivariable Cox proportional hazards model was fitted in the unmatched cohort to examine factors associated with new-onset AF. The following covariates were entered simultaneously: age per 5-year increase, sex, hypertension, chronic kidney disease, prior ischaemic stroke, socioeconomic status, residential region, beta-blocker use, and treatment group (ARNI vs. traditional RAS blockade). Mutually adjusted HRs with 95% confidence intervals (CIs) were estimated. Prespecified subgroup analyses were conducted for the primary outcome according to age, sex, socioeconomic status, region, comorbidities (diabetes mellitus, hypertension, dyslipidaemia, ischaemic heart disease, cerebrovascular disease, ischaemic stroke, peripheral artery disease, and chronic kidney disease), Charlson comorbidity index, concomitant medications, and healthcare utilization variables. Treatment-by-subgroup interactions were tested by including interaction terms in the Cox models. Subgroup and interaction analyses were considered exploratory, and no adjustment for multiple testing was applied. The final analytic dataset contained no missing values for any variables included in the analyses. Under the prespecified claims-based operational definitions, binary diagnosis, procedure, medication-use, and healthcare utilization variables were coded as present when a qualifying claim was identified and absent otherwise. Consequently, no imputation or other missing-data procedure was performed. All statistical analyses were conducted using SAS version 7.1 (SAS Institute) and R version 4.1. All tests were two-sided, and a *P* value <0.05 was considered statistically significant.

## Results

### Study population and baseline characteristics

A total of 39 061 patients with HFrEF without a history of AF were identified, comprising 12 263 patients in the ARNI group and 26 798 in the RAS blockade group (*Figure 1*). After propensity score matching, 9030 patients were included in each group. Before matching, the ARNI group was younger (66.0 ± 14.0 vs. 69.0 ± 14.8 years; ASD = 0.207), had a lower proportion of women (34.2% vs. 46.0%; ASD = 0.243), and had higher use of guideline-directed medical therapies, including aldosterone antagonists (52.0% vs. 25.1%; ASD = 0.577), beta-blockers (75.2% vs. 58.6%; ASD = 0.360), and diuretics (73.3% vs. 54.1%; ASD = 0.408). After matching, all ASDs were <0.1 (*Table 1*).

### New-onset atrial fibrillation

*Table 2* and *Figure 2* present the incidence rates and HRs for new-onset AF. In the matched cohort, new-onset AF occurred in 805 patients in the ARNI group and 880 in the RAS blockade group, with incidence rates of 10.73 and 12.15 per 100 person-years, respectively. The risk of new-onset AF was lower in the ARNI group (HR 0.89; 95% CI 0.81–0.98; *P* = 0.021). In the unmatched cohort, the adjusted HR was 0.94 (95% CI 0.87–1.02; *P* = 0.136) in Model A (all baseline covariates) and 0.91 (95% CI 0.84–0.99; *P* = 0.020) in Model B (PARADE-HF–specified covariates).

**Figure 2:**
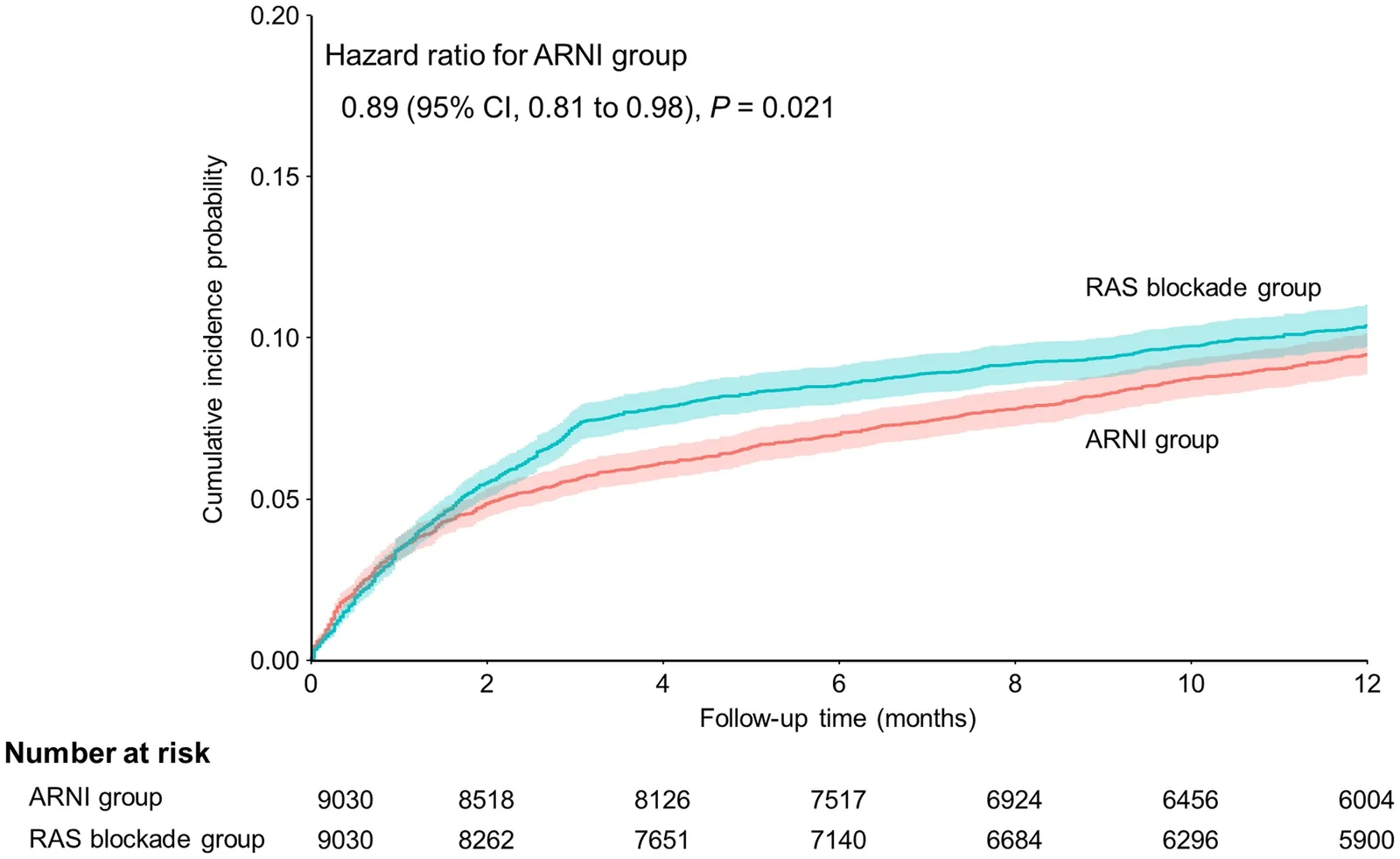
Kaplan–Meier estimates of cumulative event probability for new-onset atrial fibrillation after propensity score matching. Kaplan–Meier curves comparing the cumulative event probability of new-onset AF between the ARNI group (*n* = 9030) and the RAS blockade group (*n* = 9030) in the propensity score–matched cohort. The hazard ratio was estimated using a Cox proportional hazards model with a robust sandwich variance estimator clustered by matched pair. The number at risk is shown below the graph. ARNI, angiotensin receptor–neprilysin inhibitor; CI, confidence interval; RAS, renin–angiotensin system.

**Table 2:** Incidence rates and hazard ratios for the composite outcome, new-onset atrial fibrillation, ischaemic stroke, and all-cause death at 1 year according to treatment group (reference = RAS blockade)

	Before matching			After matching		
	RAS blockade	ARNI	*P*	RAS blockade	ARNI	*P*
Composite outcome^a^						
No. of events (IR)	4763 (21.82)	1737 (17.03)		1510 (21.06)	1330 (17.83)	
HR (95% CI)						
PSM analysis				1 (Reference)	0.85 (0.79–0.92)	<0.001
Adjusted Model A	1 (Reference)	0.89 (0.84–0.95)	<0.001			
Adjusted Model B	1 (Reference)	0.87 (0.82–0.93)	<0.001			
New-onset atrial fibrillation at 1 year						
No. of events (IR)	2849 (12.93)	1059 (10.33)		880 (12.15)	805 (10.73)	
HR (95% CI)						
PSM analysis				1 (Reference)	0.89 (0.81–0.98)	0.021
Adjusted Model A	1 (Reference)	0.94 (0.87–1.02)	0.136			
Adjusted Model B	1 (Reference)	0.91 (0.84–0.99)	0.020			
Ischaemic stroke at 1 year						
No. of events (IR)	608 (2.57)	182 (1.68)		184 (2.38)	137 (1.73)	
HR (95% CI)						
PSM analysis				1 (Reference)	0.85 (0.72–1.01)	0.063
Adjusted Model A	1 (Reference)	0.74 (0.62–0.89)	0.002			
Adjusted Model B	1 (Reference)	0.77 (0.63–0.92)	0.005			
All-cause death at 1 year						
No. of events (IR)	1949 (8.12)	710 (6.52)		633 (8.10)	558 (6.99)	
HR (95% CI)						
PSM analysis				1 (Reference)	0.86 (0.77–0.97)	0.010
Adjusted Model A	1 (Reference)	0.91 (0.83–1.00)	0.058			
Adjusted Model B	1 (Reference)	0.91 (0.83–1.01)	0.072			

Adjustment models: Model A, all baseline covariates; Model B, PARADE-HF–specified covariates.

Incidence rate is expressed per 100 person-years.

Model A included all baseline variables listed in *Table 1*. Model B followed the covariate-selection strategy used in the parent PARADE-HF study and included variables with an absolute standardized difference ≥0.1 before propensity score matching in the parent cohort. Prior AF was not included because the present cohort was restricted to patients without prior AF. The propensity score–matched estimates were obtained using Cox proportional hazards models with treatment group as the sole covariate and robust sandwich variance estimators clustered by matched pair.

AF, atrial fibrillation; ARNI, angiotensin receptor–neprilysin inhibitor; CI, confidence interval; HR, hazard ratio; IR, incidence rate; PSM, propensity score matching; RAS, renin-angiotensin system.

^a^New-onset AF, ischaemic stroke, or all-cause death.

### Secondary outcomes

The composite outcome (new-onset AF, ischaemic stroke, or all-cause death) was lower in the ARNI group in the matched cohort (HR 0.85; 95% CI 0.79–0.92; *P* < 0.001; *Table 2*). For ischaemic stroke, the point estimate in the propensity score–matched cohort was directionally consistent with the primary and composite outcomes; however, there was no clear evidence of an association (HR 0.85; 95% CI 0.72–1.01; *P* = 0.063). In the unmatched cohort, Model B (PARADE-HF–specified covariates) yielded an HR of 0.77 (95% CI 0.63–0.92; *P* = 0.005). All-cause death was lower in the matched cohort (HR 0.86; 95% CI 0.77–0.97; *P* = 0.010) (see Supplementary material online, *Figure*).

### Competing risk analysis

In the Fine–Gray subdistribution hazard regression analysis with death as a competing event, ARNI was associated with lower subdistribution hazards of new-onset AF (sHR 0.73; 95% CI 0.58–0.91; *P* = 0.005) and ischaemic stroke (sHR 0.90; 95% CI 0.82–0.99; *P* = 0.026) in the matched cohort (see Supplementary material online, *Table S2*). Before matching, the subdistribution HR for AF was 0.89 (95% CI 0.83–0.94; *P* < 0.001) in Model B (PARADE-HF–specified covariates).

### Factors associated with new-onset atrial fibrillation

In the multivariable Cox model, older age per 5-year increase (HR 1.11; 95% CI 1.10–1.13; *P* < 0.001), male sex (HR 1.22; 95% CI 1.14–1.30; *P* < 0.001), and prior ischaemic stroke (HR 1.20; 95% CI 1.08–1.34; *P* = 0.001) were associated with a higher risk of new-onset AF. ARNI use (HR 0.85; 95% CI 0.75–0.96; *P* = 0.007) and beta-blocker use (HR 0.66; 95% CI 0.62–0.71; *P* < 0.001) were associated with a lower risk (*Table 3*).

**Table 3:** Univariable and multivariable associations with new-onset atrial fibrillation in the unmatched cohort

	Univariable		Multivariable	
	HR (95% CI)	*P* value	HR (95% CI)	*P* value
Age ≥ 65 years	1.52 (1.42–1.63)	<0.001		
Age, per 5-year increase	1.10 (1.09–1.12)	<0.001	1.11 (1.10–1.13)	<0.001
Male	0.93 (0.87–0.99)	0.020	1.22 (1.14–1.30)	<0.001
Hypertension	0.71 (0.67–0.76)	<0.001	0.89 (0.83–0.95)	0.001
Chronic kidney disease	0.93 (0.82–1.05)	0.214	0.96 (0.85–1.09)	0.534
Prior ischaemic stroke	1.29 (1.16–1.43)	<0.001	1.20 (1.08–1.34)	0.001
Socioeconomic status (reference: low)				
Middle	0.97 (0.90–1.05)	0.511	0.98 (0.91–1.06)	0.678
High	0.99 (0.92–1.07)	0.784	0.92 (0.85–0.99)	0.030
Residential region (reference: rural)				
Metropolitan	0.84 (0.77–0.92)	<0.001	0.88 (0.80–0.96)	0.004
City	0.85 (0.79–0.92)	<0.001	0.91 (0.84–0.98)	0.014
Beta-blocker use	0.56 (0.52–0.59)	<0.001	0.66 (0.62–0.71)	<0.001
ARNI use	0.76 (0.70–0.81)	<0.001	0.85 (0.75–0.96)	0.007

ARNI, angiotensin receptor–neprilysin inhibitor; CI, confidence interval; HR, hazard ratio.

### Subgroup analyses

The association between ARNI use and new-onset AF was generally directionally consistent across prespecified subgroups (*Figure 3*). Nominal treatment-by-subgroup interactions were observed for hypertension, Charlson comorbidity index, peripheral artery disease, duration of prior RAS blockade, and hospitalization within 1 year; given the number of comparisons and the absence of adjustment for multiple testing, these findings should be interpreted as exploratory.

**Figure 3:**
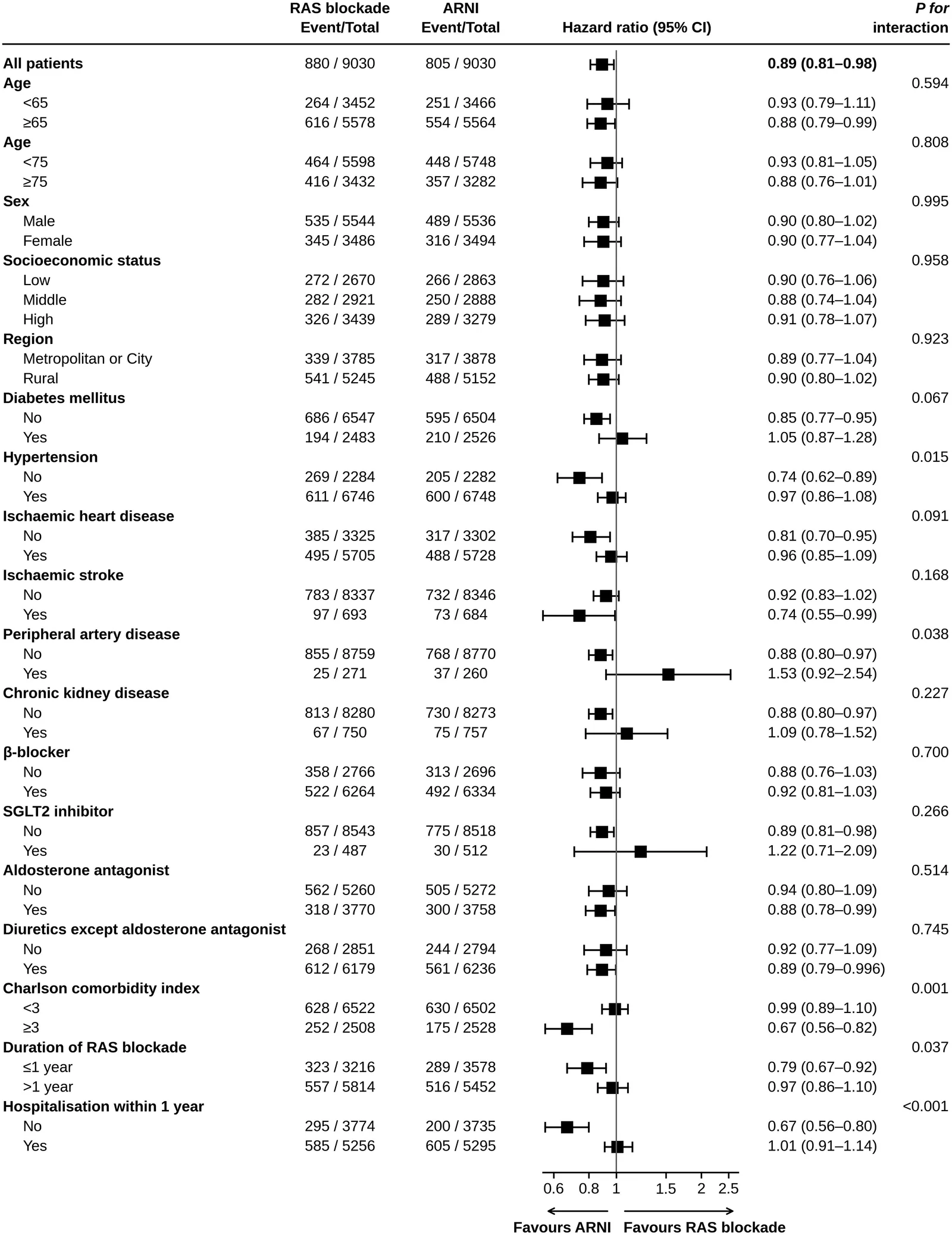
Association of ARNI with new-onset atrial fibrillation at 1 year across patient subgroups. Forest plot demonstrating the hazard ratios for new-onset atrial fibrillation in prespecified subgroups. *P* values for interaction were obtained by including treatment-by-subgroup interaction terms in the Cox models. Subgroup and interaction analyses were exploratory, and *P* values for interaction were not adjusted for multiple testing. ARNI, angiotensin receptor–neprilysin inhibitor; CI, confidence interval; HR, hazard ratio; RAS, renin–angiotensin system.

## Discussion

The main findings of this nationwide real-world study are as follows: (i) switching to ARNI was associated with a lower risk of new-onset AF than continuing traditional RAS blockade in patients with HFrEF (HR 0.89; 95% CI 0.81–0.98); (ii) this AF-preventive association was broadly consistent across propensity score matching and competing-risk analysis, although the adjusted estimate was 0.94 (95% CI 0.87–1.02) in Model A, which included all baseline covariates, and 0.91 (95% CI 0.84–0.99) in Model B, which used the PARADE-HF–specified covariates; (iii) ARNI was also associated with a reduced risk of the composite outcome of AF, ischaemic stroke, or all-cause death; and (iv) in a multivariable model, ARNI and beta-blocker use were associated with a lower risk of new-onset AF.

The association between ARNI use and a lower risk of new-onset AF observed in our study is biologically plausible. Neprilysin inhibition by sacubitril augments the endogenous natriuretic peptide system, leading to increased levels of atrial natriuretic peptide and B-type natriuretic peptide.^9^ These natriuretic peptides exert several effects that may attenuate the substrate for AF development. First, natriuretic peptides reduce left atrial pressure and volume overload by promoting natriuresis and vasodilation, thereby limiting atrial stretch and dilatation.^10,18^ Second, natriuretic peptides have anti-fibrotic and anti-hypertrophic properties mediated through cyclic guanosine monophosphate signalling, which may attenuate atrial structural remodelling.^19,20^ Third, ARNI has been shown to improve left ventricular systolic function and promote reverse cardiac remodelling, which indirectly reduces left atrial pressure.^21^

Atrial cardiomyopathy, comprising the structural, electrical, and functional atrial remodelling typically reflected in left atrial volume or diameter, is a key substrate for new-onset AF. It is mechanistically plausible that ARNI attenuates this process, as natriuretic peptide augmentation exerts anti-fibrotic, anti-hypertrophic, and favourable haemodynamic effects on the atria in addition to ventricular unloading. Consistent with this, sacubitril/valsartan has been shown to induce left atrial reverse remodelling in HFrEF, reducing the left atrial volume index and improving the left atrial emptying fraction and peak atrial longitudinal strain.^22^ These atrial effects provide a plausible mechanism linking ARNI to the lower incidence of new-onset AF observed in our study. The concept of atrial cardiomyopathy and its clinical relevance are summarized in the recent EHRA/HRS/APHRS/LAHRS clinical consensus statement.^23^ Importantly, however, neither circulating natriuretic peptide concentrations nor atrial structural measures (e.g. left atrial volume or diameter) were available in our claims-based dataset. The mechanistic links proposed above are therefore inferential and should be regarded as biologically plausible hypotheses rather than mediators established by our data.

In the PARADIGM-HF trial, new-onset AF was a prespecified secondary endpoint but showed no significant difference between sacubitril/valsartan and enalapril (84 vs. 83 events; *P* = 0.84).^6^ Meta-analyses of randomized trials, including a pooled analysis of 11 trials (11 458 patients on sacubitril/valsartan vs. 10 128 on ACE inhibitor or ARB), have likewise found no significant AF reduction, although these analyses were constrained by limited event numbers and heterogeneous HF populations.^11,12,24^ Against this background, our AF-naive HFrEF cohort accrued far more incident events and longer real-world exposure, which may explain the divergent signal. However, this remains an observational comparison: despite propensity-score matching, ARNI users represented a more intensively treated phenotype, and residual or unmeasured confounding, particularly in ventricular function, renal function, and atrial structure, not captured in claims, may account for part or all of the association. The randomized data therefore remain the higher-quality benchmark, and our findings should be regarded as complementary and hypothesis-generating rather than as overturning them.

Contemporary AF detection using opportunistic screening, wearable sensors, and implantable devices identifies considerably more AF than claims-based ascertainment and increasingly characterizes AF as a continuous burden rather than a binary present-or-absent state, an approach of particular prognostic value in HFrEF.^25^ Our dichotomous, claims-based outcome cannot capture AF burden, and future studies linking device data could refine the AF-preventive signal observed here.

Our study is a subanalysis of the PARADE-HF cohort, which showed an association between ARNI treatment and lower all-cause death and hospitalization in HFrEF; the present analysis extends that observation to new-onset AF and is likewise hypothesis-generating.^15^ Previous observational studies examining ARNI and AF were limited by small sample sizes,^26,27^ whereas our study focused exclusively on HFrEF patients without prior AF in a large nationwide cohort using multiple statistical approaches.

A particular strength of our study is the use of Fine–Gray subdistribution hazard models to account for the competing risk of death. Death as a competing event is particularly relevant in HFrEF, where high mortality can preclude AF diagnosis and confound Cox regression–based estimates of treatment association. When death was treated as a competing event using the Fine–Gray model, ARNI was associated with a lower subdistribution hazard of new-onset AF (sHR 0.73; 95% CI 0.58–0.91). Because the cause-specific and subdistribution HRs answer different questions, we report both. The cause-specific HR (0.89) reflects the association of ARNI with the rate of new-onset AF among patients remaining at risk, whereas the subdistribution HR (0.73) reflects its association with the cumulative incidence of AF when death is treated as a competing event; the two are complementary rather than interchangeable, and both indicate a lower AF risk with ARNI. All-cause death within 1 year occurred in 558 (6.2%) and 633 (7.0%) patients in the ARNI and RAS blockade groups, respectively, consistent with the parent PARADE-HF study. In the Fine–Gray analysis, ARNI use was significantly associated with a lower subdistribution hazard of ischaemic stroke (sHR 0.90; 95% CI 0.82–0.99; *P* = 0.026). However, the corresponding Cox analysis in the propensity score–matched cohort did not provide clear evidence of an association (HR 0.85; 95% CI 0.72–1.01; *P* = 0.063). Accordingly, the ischaemic stroke findings should be interpreted cautiously and considered exploratory.

Our findings have several important clinical implications. First, ARNI may provide an additional AF-preventive benefit beyond its established effects on mortality and hospitalization in HFrEF, further reinforcing guideline recommendations of ARNI as first-line therapy.^7,8^ Second, ARNI and beta-blocker use were associated with a lower risk of new-onset AF in the multivariable analysis, supporting further prospective evaluation of their potential roles in AF prevention.^28–30^ These findings align with previous real-world studies reporting favourable clinical outcomes with ARNI.^31,32^ Third, the competing risk–adjusted analysis showed an association between ARNI use and a lower subdistribution hazard of ischaemic stroke, although whether this finding reflects a downstream clinical benefit related to AF prevention remains uncertain. Fourth, nominal interactions were observed in several prespecified subgroups; however, because no adjustment for multiple testing was performed, these exploratory findings should not be interpreted as definitive evidence of heterogeneity in the treatment association and require confirmation in prospective studies.

Several baseline associations in the multivariable model (*Table 3*) warrant cautious interpretation. Notably, hypertension was associated with a lower risk of new-onset AF, the opposite of its established role in the general population. This is consistent with the ‘blood-pressure paradox’ of heart failure, in which higher blood pressure predicts better outcomes; in OPTIMIZE-HF, higher admission systolic blood pressure was associated with progressively lower mortality irrespective of ejection fraction.^33^ In established HFrEF, a hypertensive phenotype likely reflects greater contractile reserve and less advanced atrial remodelling, behaving as a marker of less severe disease rather than a causal AF risk factor; index-event (collider) bias from conditioning on HFrEF may further reverse such associations. These within-HFrEF associations should not be extrapolated to unselected populations.

This study has several limitations. First, as an observational analysis, it is subject to residual and unmeasured confounding that propensity-score matching cannot eliminate, and no per-protocol analysis was performed; the findings are therefore hypothesis-generating rather than confirmatory. Second, claims data lack several prognostically important variables, including laboratory values (and thus renal function and left ventricular ejection fraction), New York Heart Association class, and echocardiographic measures including left atrial size, a powerful predictor of incident AF, leaving these as unmeasured confounders; this is particularly relevant for renal function, given the differential estimated glomerular filtration rate thresholds for ARNI vs. ACE inhibitors or ARBs. Third, AF was ascertained from claims, which capture neither symptom status nor monitoring intensity; because asymptomatic AF is common in HFrEF,^34^ our outcome probably underestimates the true incidence and may be subject to ascertainment bias. Fourth, the HFrEF definition was not fully symmetrical between groups (partly reimbursement-based for ARNI, claims-plus-testing-based for RAS blockade), which may introduce misclassification. Fifth, selection bias is likely: the ARNI group was more intensively treated, and eligibility required prior RAS tolerance, as in PARADIGM-HF.^6^ By design the cohort therefore comprised prevalent patients with long-standing HFrEF (mean prior RAS-blockade duration >3 years), frequent periodic outpatient follow-up, and no documented AF over more than 3 years of prior observation, identifying a relatively low-risk, treatment-adherent population; this selection, together with the exclusion of early deaths and of patients without at least 90 days of therapy, probably contributed to the comparatively low 1-year mortality. Sixth, RAS blockade was analyzed as a single comparator class without agent-level comparison, and ventricular arrhythmias were not assessed. Finally, generalisability is constrained on several counts: the cohort was Korean,^35^ follow-up was limited to 1 year, sodium-glucose cotransporter-2 inhibitors, now recognized as potentially AF-preventive, were seldom used during the study period (2017–2021),^36^ and the low-risk, prevalent-user nature of the cohort limits applicability to RAS-naive, RAS-intolerant, newly diagnosed, less closely followed, or sicker patients; whether the association holds across other populations, longer horizons, and contemporary therapy remains to be established.

## Conclusions

In this large, real-world cohort of AF-naive patients with HFrEF, switching to ARNI was associated with a lower risk of short-term new-onset AF than continuing traditional RAS blockade. As this was a retrospective study, further prospective studies are needed to confirm these findings.

## Supplementary Material

pvag074_Supplementary_Data

## Data Availability

Data sharing by the authors is not possible because of the Korean Government regulations. However, additional data are available through approval and oversight by the Korean National Health Insurance Service.
