# Supplementary material for: Switching to angiotensin receptor–neprilysin inhibitor and reduced risk of new-onset atrial fibrillation in heart failure with reduced ejection fraction: a nationwide propensity score–matched study
Source: Eur Heart J Cardiovasc Pharmacother. 2026 Sep 15;12(7):535–45. doi: 10.1093/ehjcvp/pvag074 (PMC13730560; doi:10.1093/ehjcvp/pvag074)
Supplement: Supplementary file 1 [file pvag074_supplementary_data.docx]

Supplementary Table 1. Individual agents comprising traditional RAS blockade

**RAS blockade**

candesartan

candesartan+amlodipine

candesartan+hydrochlorothiazide

captopril

cilazapril

enalapril

eprosartan

eprosartan+hydrochlorothiazide

fimasartan

fimasartan+amlodipine

fimasartan+hydrochlorothiazide

imidapril

irbesartan

irbesartan+hydrochlorothiazide

lisinopril

losartan

losartan+amlodipine

losartan+chlorthalidone+amlodipine

losartan+hydrochlorothiazide

olmesartan

olmesartan+amlodipine

olmesartan+hydrochlorothiazide

olmesartan+hydrochlorothiazide+amlodipine

perindopril

perindopril+indapamide

ramipril

ramipril+felodipine

telmisartan

telmisartan+amlodipine

telmisartan+chlorthalidone+amlodipine

telmisartan+hydrochlorothiazide

telmisartan+hydrochlorothiazide+amlodipine

valsartan

valsartan+amlodipine

valsartan+hydrochlorothiazide

valsartan+lercanidipine

zofenopril

RAS = renin-angiotensin system.

Supplementary Table 2. Fine–Gray subdistribution hazard regression for new-onset atrial fibrillation and ischaemic stroke with death as the competing event (reference = traditional RAS blockade)

Adjustment models: Model A, all baseline covariates; Model B, PARADE-HF–specified covariates.

|  | Before PSM **sHR (95% CI)** | *P* | After PSM  **sHR (95% CI)** | *P* |
| --- | --- | --- | --- | --- |
| Atrial fibrillation |  |  |  |  |
| PSM Fine–Gray results |  |  | **0.73 (0.58–0.91)** | **0.005** |
| Adjusted Model A | **0.91 (0.86–0.97)** | **0.005** |  |  |
| Adjusted Model B | **0.89 (0.83–0.94)** | **<0.001** |  |  |
| Ischaemic stroke |  |  |  |  |
| PSM Fine–Gray results |  |  | **0.90 (0.82–0.99)** | **0.026** |
| Adjusted Model A | **0.87 (0.79–0.95)** | **0.001** |  |  |
| Adjusted Model B | **0.87 (0.79–0.95)** | **0.002** |  |  |

Subdistribution hazard ratios were estimated using Fine–Gray regression with death as the competing event; traditional RAS blockade was the reference group. Model A and Model B were applied only to analyses of the unmatched cohort. Model A included all baseline variables listed in Table 1. Model B followed the covariate-selection strategy used in the parent PARADE-HF study and included variables with an absolute standardised difference ≥0.1 before propensity score matching in the parent cohort.

PSM, propensity score matching.

Supplementary Figure. Cumulative incidence of composite outcome, ischaemic stroke, and all-cause death (after matching)
